# Building a World-ship: A decolonial approach to just energy transitions

**DOI:** 10.1177/19427786251326605

**Published:** 2025-03-19

**Authors:** Rebeca Roysen, Guilherme Moura Fagundes, Lasse Kos, Nadine Bruehwiler, Jens Koehrsen

**Affiliations:** 1 27209 Center for Religion, Economics and Politics, University of Basel, Basel, Switzerland; 2 28133 Department of Anthropology, University of Sao Paulo, Sao Paulo, Brazil; 3 6305 Faculty of Theology, University of Oslo, Oslo, Norway

**Keywords:** grassroots innovations, energy transitions, decolonial ecology, just transitions, ecovillages, Global South, commons, quilombos, innovaciones de base, transiciones energéticas, ecología decolonial, transiciones justas, ecoaldeas, Sur Global, bienes comunes, quilombos

## Abstract

This article contributes to discussions on just energy transitions by analyzing a community project in the slums of Sao Paulo, Brazil. Instituto Favela da Paz (IFDP) is an urban ecovillage created by slum-dwellers who develop projects and courses on renewable energies, urban permaculture, healthy food, and arts. We draw upon decolonial ecology framework (DEF) developed by Caribbean scholar Malcom Ferdinand to analyze their innovations. Based on participatory network mapping, participant observations, interviews, and focus groups, we address the following research questions: In what ways does IFDP promote just energy transitions? And how can the DEF help us understand these types of grassroots innovations that are emerging in the peripheries of the Global South? Instituto Favela da Paz promotes just energy transitions through the dissemination and democratization of renewable technologies, and by reframing narratives that keep in place the exploitation of cheap labor, especially from racialized groups. Instituto Favela da Paz and its partners engage in activities that reframe meanings associated with time, energy, money, and Black identity through their practices of *aquilombamento.* We conclude that the DEF enriches the theoretical tools available to analyze just energy transitions by bringing to the forefront the exploitation of human body energy—be it in the form of slavery, cheap labor, or gender inequality—that maintains unsustainable ways of inhabiting the Earth. It also helps to identify the emergence of alternative ways of living that contribute to the creation of a more just and shared world.

## Resumen

Este artículo contribuye a las discusiones sobre las transiciones energéticas justas mediante el análisis de un proyecto comunitario en las favelas de Sao Paulo, Brasil. El Instituto Favela da Paz (IFDP) es una ecoaldea urbana creada por habitantes de favelas que desarrollan proyectos y cursos sobre energías renovables, permacultura urbana, alimentos saludables y artes. Nos basamos en el marco de la ecología decolonial desarrollado por el académico caribeño Malcom Ferdinand para analizar sus innovaciones. Con base en el mapeo participativo de redes, observaciones participantes, entrevistas y grupos focales, abordamos las siguientes preguntas de investigación: ¿De qué manera promueve el IFDP las transiciones energéticas justas? ¿Y cómo puede la ecología decolonial ayudarnos a comprender este tipo de innovaciones de base que están surgiendo en las periferias del Sur Global? El IFDP promueve transiciones energéticas justas a través de la difusión y democratización de tecnologías renovables y reformulando las narrativas que mantienen vigente la explotación de la mano de obra barata, especialmente de los grupos racializados. El IFDP y sus socios participan en actividades que reformulan los significados asociados con el tiempo, la energía, el dinero y la identidad negra a través de sus prácticas de aquilombamento. Concluimos que la ecología decolonial enriquece las herramientas teóricas disponibles para analizar las transiciones energéticas justas al poner en primer plano la explotación de la energía del cuerpo humano (ya sea en forma de esclavitud, mano de obra barata o desigualdad de género) que mantiene formas insostenibles de habitar la Tierra. También ayuda a identificar el surgimiento de formas alternativas de vida que contribuyen a la creación de un mundo más justo y compartido

## Introduction

The global risks associated with climate change are now widely acknowledged (IPCC, 2023; Pörtner et al., 2022). National governments and corporations are investing in the so-called “energy transition” in order to mitigate the risks associated with global warming above 1.5° compared to preindustrial levels. These discussions on energy transitions tend to focus on the change of energy sources from high-carbon-emitting to lower-carbon-emitting ones. However, mainstream solutions to energy transitions continue to reproduce and expand capitalism and its injustices (Dunlap and Tornel, 2023; Lang et al., 2024; Siamanta, 2024; Sovacool, 2021). It is in this context that the literature on just energy transitions has emerged. Scholars in this field link the transition agenda to social justice concerns (Schlosberg and Collins, 2014; Williams and Doyon, 2019), while some also envision post-capitalist and decolonial strategies for energy transitions (Dunlap and Tornel, 2023; Siamanta, 2024; Swilling, 2019; Velasco-Herrejón et al., 2022).

This article contributes to the literature on just energy transitions by engaging with decolonial ecology framework (DEF) developed by the Caribbean scholar Ferdinand (2022). This framework understands the degradation of the environment and the degradation of a vast part of the global population as two sides of the same phenomenon: the colonial way of inhabiting the Earth. Ferdinand (2022) calls for ways to face current problems in which the plurality of humans and nonhumans have the conditions to act together, from their embodied experience in the world. He calls this process of political encounter the building of a World-Ship.

We use Ferdinand's concepts to analyze a grassroots project that emerged in the slums of Sao Paulo, Brazil. Instituto Favela da Paz (IFDP) is an urban ecovillage^1^ created by slum-dwellers who develop projects and courses on renewable energies, urban permaculture, healthy food, and arts. In this article, we address the following research questions: In what ways does IFDP promote just energy transitions? And how can the DEF help us understand these types of grassroots innovations that are emerging in the peripheries of the Global South? As we demonstrate throughout the article, the DEF, in thinking the exploitation of nature and the exploitation of labor as two sides of the same phenomenon, helps us to think about energy not only as electricity, heat, or fuel but also as human body energy. In this sense, the exploitation of human energy follows the same core-periphery pattern as the exploitation of other forms of energy. This lens enables the understanding of IFDP as an initiative of just energy transition: not only because of their community-owned solar energy technologies but mainly because of their shared economy and sociocultural activities, which help to reframe meanings associated with the role of peripheral actors in the city of Sao Paulo.

This article is structured as follows: First, we present the literature on just energy transitions, and the decolonial ecology framework. After describing the methods used and introducing the context of IFDP, we present our results. In the discussion section, we reflect on the ways in which the DEF and the empirical data from the case study can help us understand just energy transitions from a broader perspective. In the conclusion section, we reiterate our contribution to current discussions of justice in energy transitions.

## Theoretical framework

### Just energy transitions

There is a growing literature on *just* energy transitions. Part of this literature focuses on just transitions as a labor-oriented concept, for example, by mitigating economic hardships in fossil fuel-dependent communities and encouraging job generation in the field of renewable energy (Snell, 2018; Stevis and Felli, 2015; Wang and Lo, 2021). However, this understanding of just transition is based on the maintenance of an economic system that, while redistributing compensations, can only continue to thrive by producing low-income working-class groups. “In this respect, justice principles may actually reinforce rather than disrupt consensual thinking about trickle-down economics” (Velicu and Barca, 2020, p. 265).

A more critical line of work, in which we position our article, grounds the concept of just transitions in the environmental justice movement, and the struggles of vulnerable groups that are disproportionately impacted by environmental degradation (Schlosberg and Collins, 2014; Wang and Lo, 2021; Williams and Doyon, 2019). They link the transition agenda to other social justice concerns such as the indigenous land rights movement, economic justice movements, and civil rights groups. By historicizing energy practices, critical scholarship shows that socioecological inequalities are not only the “impact of” but also “inputs to” dominant energy systems which continue to depend on colonialism, racism, and differences in gender and class (Partridge, 2022; Tornel, 2023).

Many scholars from this critical line of work see the need for a radical reconfiguration of current power relations, including a reduction in the powers and wealth of the rich and super-rich (Swilling, 2019). An Oxfam report (Khalfan et al., 2023) shows that the richest 1% of the world's population is responsible for as much carbon pollution as the people who make up the poorest two-thirds of humanity. Despite being those least responsible for pollution, the poor tend to suffer the most in energy transition efforts. A recent review of 332 cases has shown that, more often than not, efforts to facilitate energy transitions tend to exacerbate social vulnerability, discrimination, exclusion, and the concentration of wealth (Sovacool, 2021). There is an increased understanding that many actions involved in scaling up renewable energy production and the adoption of electric vehicles and batteries create unintended and unjust consequences for marginal communities (Bathke, 2014; Dunlap and Riquito, 2023; Hernandez and Newell, 2022; Joshi and Kothari, 2024; Kumar et al., 2021; Lang et al., 2024; Partridge, 2022; Siamanta and Dunlap, 2019).

Critical scholarship on energy transitions from a geographical perspective has linked transitions to preexisting processes of spatial differentiation and uneven geographical development (Golubchikov and O'Sullivan, 2020; O'Sullivan et al., 2020). Spatial differentiation is shaped by physical, historical, cultural, economic, and political conditions which create peripheral regions at the international, national, and regional levels. Core-periphery development is recognized as inherent to capitalism, which unevenly distributes political, economic, and cultural forces within society, creating places of exploitation and marginalization (O'Sullivan et al., 2020).

While peripheral places described in the literature are usually (rural) areas rich in natural resources and energy producers, urban peripheries in the Global South are also places of labor reserve that feed the cities’ “core.” In Brazil, for example, the emergence of poor urban peripheries, especially after the 1950s, was the result of different socioeconomic developments, in particular, the increased industrial activity in the urban space (Caldeira, 2017; Kowarick, 2009). This activity required cheap labor and attracted many people fleeing from the instabilities of rural areas and in search of better conditions of life. These migration movements, however, were not met with social rights, housing policies, and urban planning, leading migrants to look for cheap (or illegal) land, far from the city centers, where they could build their own houses (Fernandes and Gama-Rosa Costa, 2012; Kowarick, 2009). This sprawl of poor urban peripheries has led to a “tremendous gap between poor spaces with precarious or inexistent urban infrastructure and the city center where jobs and cultural and economic opportunities are concentrated” (Rolnik, 2001, p. 471).

Urban peripheries in Brazil are extremely heterogeneous in terms of urban and housing conditions, and with regard to socioeconomic conditions of their dwellers (Caldeira, 2017). In general, residents of peripheries have restricted access to systems of energy, water, sewage, urban mobility, and cultural offerings, in addition to being subjected to social stigma (Kowarick, 2009). These urban peripheries can be understood as energy peripheries (Golubchikov and O'Sullivan, 2020) since local residents suffer disadvantages in the generation, distribution, and consumption of energy. They cannot afford the costs of installing decentralized energy technologies, and their houses are usually of low-quality and with low-energy efficiency. High-energy prices in relation to household incomes also make them more vulnerable to energy poverty (Bouzarovski and Simcock, 2017).

Some authors have suggested that the idea of a just transition has been appropriated by corporations and governments who have reduced it down to pricing schemes and technomanagerial approaches, leaving capitalism unquestioned (Bouzarovski, 2022; Bringel and Fernandes, 2024). As an alternative, many scholars are looking at low-tech community-based solutions that focus on well-being rather than on economic growth, and noncapitalist relations of production, ownership, exchange, and circulation (Höffken et al., 2021; Melnyk and Singh, 2021; Siamanta, 2024; Swilling, 2019).

However, the literature available on grassroots energy innovations comes mostly from studies of initiatives in the Global North (e.g., Brummer, 2018; De Vries et al., 2016; Martiskainen, 2017; Rogers et al., 2012; Seyfang et al., 2013). There are very few documented cases of grassroots energy innovations created *by and for marginalized groups* in the Global South (see, e.g., Avila-Calero, 2017; Dunlap, 2018; Lai, 2023; Lang, 2024). In her study, for example, Lai (2023) shows how a community energy project initiated by victims of industrial pollution in Taiwan became a symbol of the community's agency and hope, as they managed to reframe their degraded sense of place into a place-based vision of sustainability. According to Lai, research on grassroots innovations tends to focus too much on the sociotechnical systems approach, largely overlooking grassroots aspirations and visions beyond the sociotechnical domain. She then suggests a more justice-sensitive approach to studying grassroots innovations, especially those arising in marginalized settings: an approach that also looks at their transformative potential for addressing structural injustices and for creating place-based visions of sustainability (Lai, 2023).

In this article, we add to the sparse literature on grassroots energy innovations in marginalized communities in the Global South by documenting the case study of a community project that emerged in the slums of Sao Paulo, Brazil. In order to understand their transformative potential and place-based visions, we first introduce some concepts from Ferdinand's DEF.

### Decolonial ecology

The DEF is relevant for discussions on energy transitions in three main ways. Firstly, it helps to understand the exploitation of nature and of human beings as two sides of the same phenomenon: *the colonial inhabitation*. The colonial inhabitation is a violent way of inhabiting the Earth that has led to the genocide of indigenous peoples, the destruction of ecosystems, the transatlantic slave trade, the exploitation of nature, and the transformation of territories into plantations. The colonial inhabitation has three main principles: (1) It creates economic and ontological dependencies between geographical places (e.g., colonies and metropolises); (2) It is based on the exploitation of land, humans, and nonhumans, treated as “resources” for commercial purposes and for the enrichment of a few; and (3) The refusal of the Other, of a person who is different in appearance, belongings, and beliefs (Ferdinand, 2022).

Secondly, the DEF helps to understand “energy” not only as electricity, heat, or fuel but also as the human body energy that is necessary to keep the status-quo of growth-oriented capitalist societies. Since the 15th century, the establishment of plantations in the Americas was made possible through the violent slave-ship device, where Black bodies were chained in the hold of the ship. This “hold politics” deprived humans and other living beings of their own worlds^2^ and reduced them to the only purpose of exploiting their work and bodies. Black people enslaved to work at the American plantations were also called “ebony wood,” revealing how they were discursively transformed into renewable “energy resources” through the transatlantic slave trade and the politics of natality. Ferdinand uses the word *nègre* (translated to English as Negro) not as a synonym for race or for the color of the skin. It is a word that defines all those who were—and still are—in the hold of the modern world: who are excluded from it, and reduced to their energy value.

Parallels between the exploitation of energy sources and the exploitation of human bodies were also established by other authors (Fiori, 2020; Mouhot, 2011; Nikiforuk, 2012). This line of research has identified a correlation in time and space between the advent of the steam engine and the abolitionist movement. The advent of machines run by fossil fuels created a new surplus of power. This led to a growing belief that technical progress would eliminate the need for unpleasant work (Mouhot, 2011). However, the transition from slavery to carbon-based machines did not imply a rupture but the continuation of a system of values that depended on the concentration of cheap surplus energy (Nikiforuk, 2012). In fact, at the turn of the 19th century, the adoption of machines in the plantations led to profound changes in the exploitation of Black bodies. These changes were “marked by the incorporation of industrial production techniques and the development of labor discipline that revitalized the institution of slavery” (Fiori, 2020, p. 565). Black labor was then reframed as one element of the mechanical processes of production—one which required management and disciplining (Fiori, 2020).

The colonial inhabitation is expressed today in factories, in instances of land grabbing, deforestation, in racial and misogynistic hierarchies, in natural parks that expulse local communities, and in various other forms of construction of “lands without a world” (Ferdinand, 2022). The slave quarters of today are expressed in the urban differences between habitations and shacks as well as in the “daily material and energetic procession of all of the Earth's Negroes, going from their shacks to the masters’ habitation” (Ferdinand, 2022, p. 61). In Brazil, this is visible in the unequal spatial development of the cities as well as in the types of jobs available to different sectors of the population. Poorer and racialized workers are more likely to be subject to precarious jobs with longer working hours, degrading working conditions, and other forms of workspace violence, such as humiliation and burnout (see, e.g., Franco et al., 2010; Vieira, 2023). Colonial inhabitation is a system in which “a minority feeds upon the vital energy of a majority that is socially discriminated against and politically dominated” (Ferdinand, 2022, p. 59).

This domination, however, has always faced different forms of resistance which can enrich our conceptual tools to think about the ecological crisis. One of the most potent forms of resistance is the marronage (Ferdinand, 2022) or, in Portuguese, the *aquilombamento*, that is, the creation of *quilombos*. Historically, in Brazil, the term *quilombo* (of Bantu linguistic origin) refers to communities established by Africans who escaped plantations in the slavery period. During the conception of the federal constitution of 1988, an articulation of anthropologists and legal experts enabled a resemantization of the term *quilombos* as a category for recognizing specific rights for these traditional communities of African descent (including collective ownership of land) (Fagundes, 2022). However, this term is also reclaimed in a contemporary fashion by Black communities in both rural and urban contexts as a form of resistance and belonging (see, e.g., Fagundes, 2022; Henson, 2024; Montrazio and Silva, 2023; Morais, 2023; Paula and Lucio, 2022; Sena and Risso, 2023; Valois, 2019). In Ferdinand's work, *aquilombamento* is an experience of emancipation, of rediscovery of cultural belongings and recreation of collective forms of living. “Faced with a colonial inhabitation that is devouring the word, the Marroons [or *quilombolas*] put into practice another way of living together and relating to the Earth” (Ferdinand, 2022, p. 147).

Finally, the third way in which the DEF is relevant for discussions on energy transitions is that it sees the imaginary as a fundamental aspect of ecological struggles. Ferdinand suggests the image of a “World-Ship” to overcome the imaginaries of both “Noah's Ark” and the “Slave-Ship.” While the imaginary of “Noah's Ark” produces a “politics of embarkation” in which few are chosen for salvation from the environmental catastrophe, the “Slave-Ship” produces a “politics of disembarkation” in which racialized people are conceived outside ecological relationships. Finally, the image of the “World-Ship” in Ferdinand's work represents the “politics of encounter” among the diversity of humans and nonhumans to face the storm (climate change) together. The “World-Ship” is about building relationships and alliances, including those who were previously relegated to the hold of the slave-ship, but who now join the decks and become shipmates in a common World-Ship (Ferdinand, 2022). This concept thus proposes an epistemic shift in current understandings of just transitions by including the visions and solutions developed by historically marginalized groups in ongoing transitions.

## Methods

This study is part of the research project Ecovillages as Incubators for Sustainability Transitions (EVIST). The first author conducted fieldwork at IFDP between November 6 and December 4, 2023. It consisted of participatory observations, two focus groups (one at the beginning of fieldwork and one to discuss these results^3^), and 13 interviews with members of the ecovillage as well as with external actors, such as collaboration partners and locals who participated in courses at the ecovillage. We also included an exploratory interview in the analysis, which was conducted with one of the founders of the ecovillage during the first year of the research project.

Interviews included questions about the perceived differences, relationships, and collaborations between ecovillage members and other people from the ecovillage's local social environment, as well as questions about the local impact of the ecovillages’ innovation activities. All interviews were transcribed, translated, and analyzed with the support of DeepL Pro and MAXQDA (VERBI Software, 2024). Additional information from the interviewees was also collected via digital communication throughout the year of 2024.

The names of all interviewees were changed in order to protect their identities. Decisions on which external actors to interview were based on field observations and on a participatory network mapping (PNM), loosely inspired by the work of Öztekin (2022). The PNM was conducted after the first focus group and consisted of the identification and drawing of the different collaboration partners of the ecovillage. The decision for the PNM was based on the premises of decolonial methodologies which avoid imposing the scientific and Eurocentric gaze on marginalized groups. On the contrary, decolonial methodologies consider these groups as agents and active participants of the research process (Faria, 2018). Information on the interviews can be found in Table 1.

**Table 1.** Information on the interviews analyzed in this study.

Interview code	Codename of interviewee	Name of organization	Format	Date of interview
EI09	Vini	IFDP (cofounder)	Zoom	29 and 30.04.2022
FP01	Jorge	Samba na 2/ Neighbor of IFDP	Zoom	05.10.2023
FP02	Focus Group with Alice (resident), Beth (nonresident member and neighbor), Vini (cofounder), Marcela (recent resident) and Mirela (resident).	IFDP	In person	10.11.2023
FP03	Alice	IFDP (resident)	In person	13.11.2023
FP04	Mirela	IFDP (resident)	In person	14.11.2023
FP05	Ney	IFDP (nonresident member)	In person	15.11.2023
FP06	Lucas	IFDP (resident)	In person	16.11.2023
FP07	Eduardo	Student/ photographer	In person	19.11.2023
FP08	Amanda	Worley Engenharia	Zoom	20.11.2023
FP09	Keto	Espírito de Zumbi - Instituto de Culturas Afrobrasileiras	In person	22.11.2023
FP10	Douglas	Independent artist	In person	22.11.2023
FP11	Kelly	Instituto Coração da Portela	In person	23.11.2023
FP12	Fabiano	Student/ electrician	In person	28.11.2023
FP13	Vera	IFDP (cofounder)	In person	28.11.2023
FP14	Veronica	Unikebradas (Unidiversidade das Quebradas)	Zoom	01.12.2023

The names of all interviewees were changed in order to protect their identities.

Some limitations of this study are the short research stay, and the primary focus of the research on the external activities of the ecovillage. The field researcher did not have the chance to participate in internal meetings, nor to capture internal power dynamics among the members of the ecovillage. On the other hand, the researcher participated in several activities where ecovillage members interacted with neighbors and external partners (such as courses, cultural activities, and events), and collected observation data in order to triangulate interview data on the impact of the ecovillage in the slum where they are located. Before we present our results, in the next subsection, we briefly describe the context in which IFDP emerged.

### The context: Jardim Ângela district, Sao Paulo city

Between 1930 and 1980, Brazil witnessed a massive displacement of inhabitants from rural areas and small urban agglomerations toward the large Brazilian metropolises, especially to the Greater Sao Paulo area. These migrants were inserted into the productive machinery and had the possibility of integration into the city through self-building houses in peripheral places, which were slowly connected to basic urban services (Caldeira, 2017; Kowarick, 2009; Rolnik, 2001). Over time, the expansion of autonomous and informal work, the widespread outsourcing dynamics with the consequent reduction in permanent and regular wage earners, and the growth of the reserve army of labor led to widespread social and economic vulnerability (Kowarick, 2009).

Around the mid-1970s, in the municipality of Sao Paulo, slum-dwellers represented only 1% of the population (around 72,000 people). In 1980, the number rose to 4.4% (more than 800,000), and in 2000, it reached 11.2% (1,610,000) (Kowarick, 2009). Today, people that live in peripheries and slums (in Brazil, also called *periferias*, *comunidades*, *favelas*, or *quebradas*) account for around 8.1% of the Brazilian population (16.4 million people) (Bello, 2024). They live in a situation of socioeconomic and civic vulnerability related to their rights to housing, health, sanitation, education, and police protection for their physical integrity (Kowarick, 2009). Their living conditions express the *social apartheid* of Brazilian cities (Kowarick, 2009; Lemos, 2023), which excludes and denies rights to those that are perceived as different or inferior, and who are associated in the social imaginary with poverty and criminal behavior.

**Figure 1.**
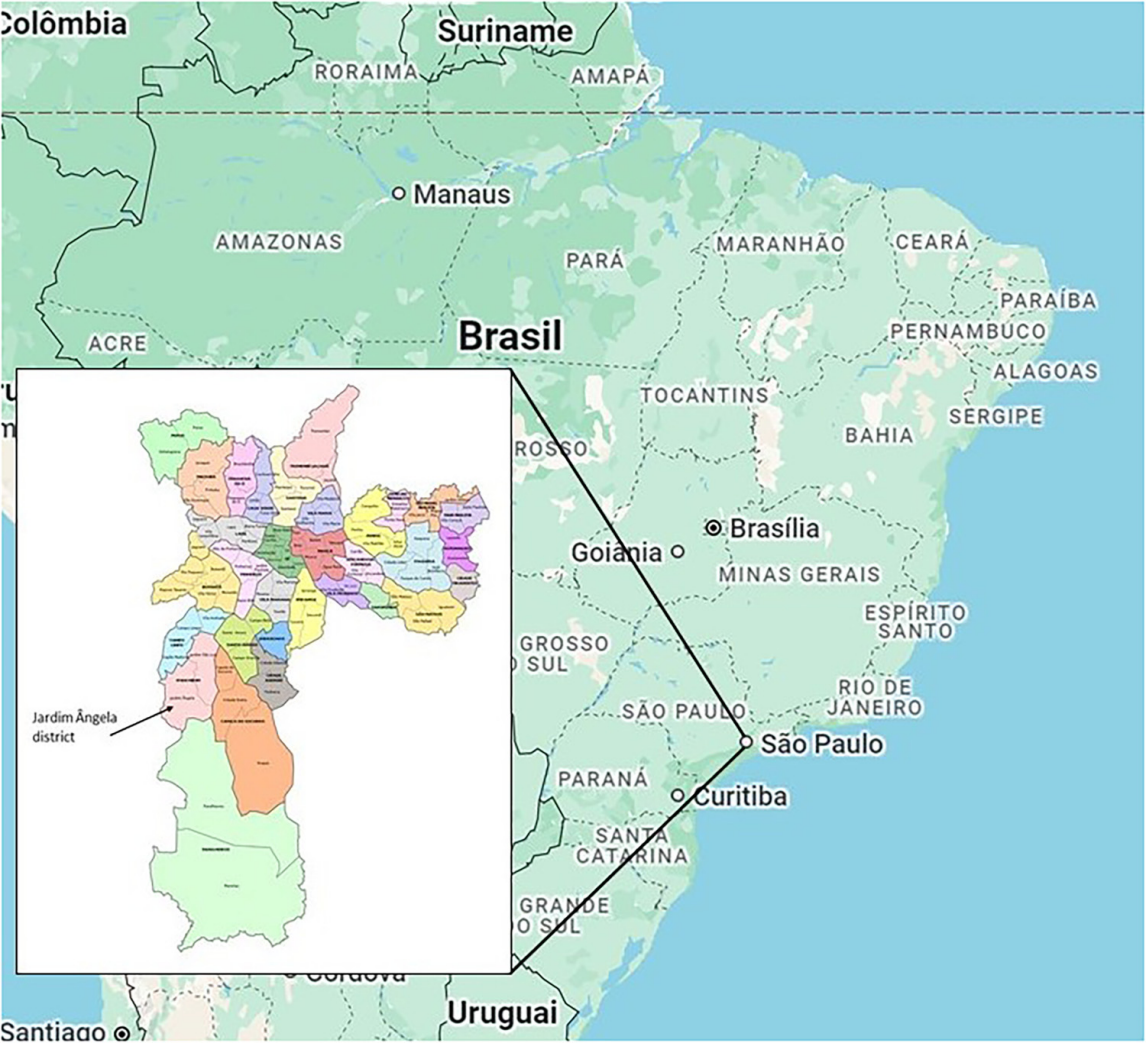
Jardim Ângela district in the municipality of Sao Paulo, and its location on the Brazilian map. Organized by the authors.

*Sources:* Wikipedia Commons (n.d.) and Google Maps.

**Figure 2.**
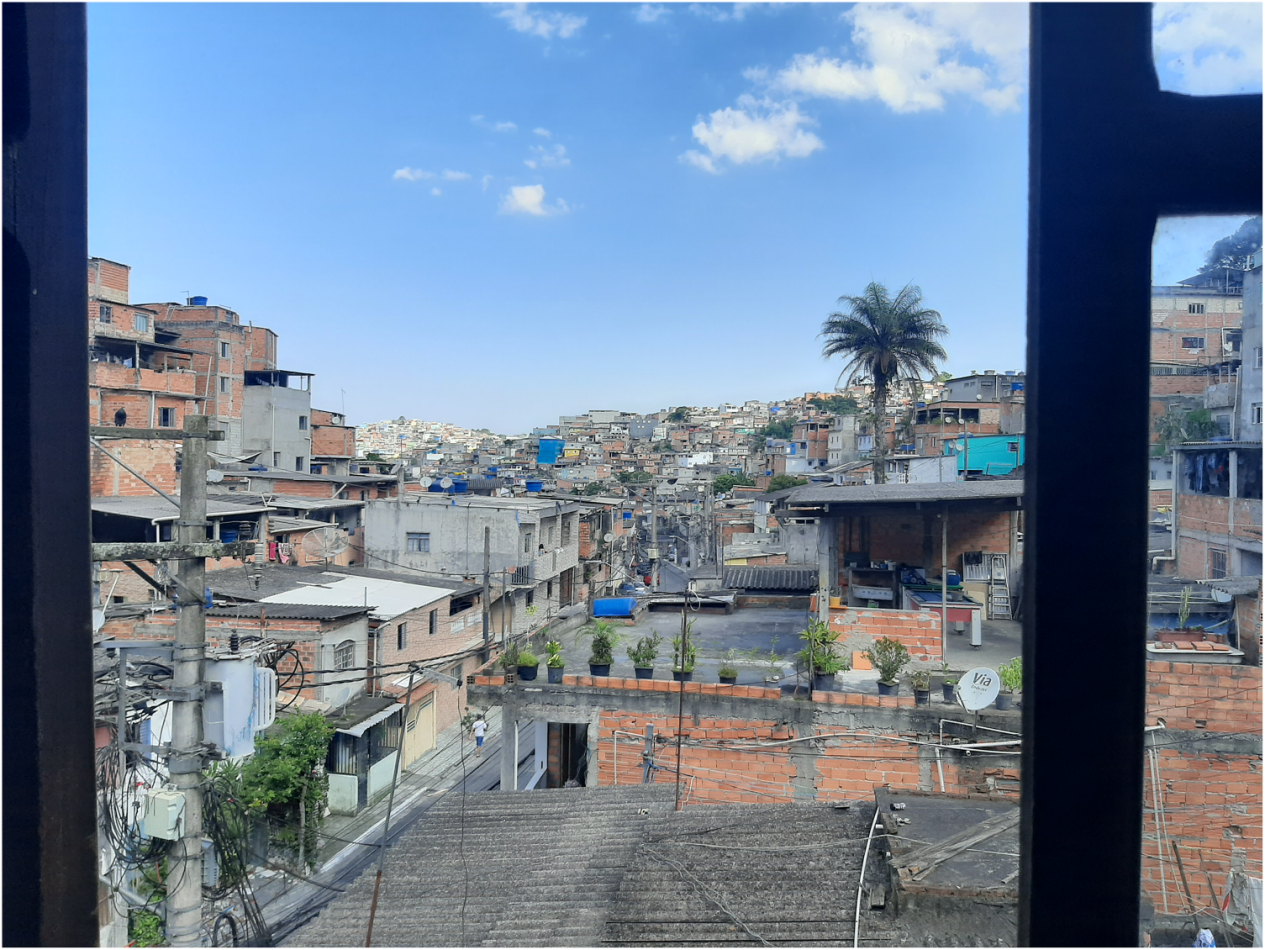
View of Jardim Ângela from a window of IFDP.

*Source:* First authors’ archive.

Jardim Ângela is a peripheral district in the southern zone of Sao Paulo (Figure 1), with a population of 345,530 people living in its 37.4 km^2^. It is the second most populous district in the city (Figure 2). The uneven spatial development of the city is expressed in the performance of Jardim Ângela (in terms of conditions of housing, health facilities, education, sports, mobility, culture, security, work, and income), which was ranked 90^th^ place (out of 96 districts) by the Inequality Map 2023 (Mapa da Desigualdade, 2023). According to Mapa da Desigualdade (2023), in the district of Jardim Ângela, the offer of formal employment is 0.5 jobs per 10 inhabitants. Jardim Ângela also has the second lowest score in individual micro-businesses. This means that the majority of the working population have to either commute to more central neighborhoods for formal employment or work in informal (undocumented) jobs. The average time spent in commuting (excluding the walk to and from the bus/train stop) is 62-min in the morning (Mapa da Desigualdade, 2023).

In Sao Paulo, uneven development also has a strong racial component, which is expressed in the racial distribution of the population between core and peripheral areas. Jardim Ângela is the district with the highest percentage of Black population in the city (60.1%), whereas the upper-class district of Moema, for example, in a more central location, has only 5.8%. While residents of Jardim Ângela are expected to live until the age of 62, the residents of Moema tend to live until the age of 81 (Mapa da Desigualdade, 2023; Patriarca, 2022; Ramos, 2022). In 2014, the number of young men (between 15 and 29 years old) dead by homicide was 79.6 per 100,000 in Jardim Ângela and zero in Moema (Rede Nossa São Paulo, 2017a, 2017b).

## Results

In this section, we introduce the case study, followed by the four main ways through which they promote just energy transitions.

### Brief history of the case study

Instituto Favela da Paz is an urban ecovillage located in Jardim Ângela, a district in the periphery of Sao Paulo. As many other thousands of people, Rubens (the father of three members of the ecovillage's core group) migrated, around 1970, from a rural community in the northeastern part of Brazil to Sao Paulo in search of a better life. His own father was already living in the district where the ecovillage is located today. At that time, it was still a very rural area with only a few houses. Soon he started to work with his father as a mason helper until he got some jobs taking care of private swimming pools. After some years, he was able to buy a small plot in the neighborhood and to build the house where the ecovillage is today. Over time, he saw more and more people occupying the land around him, cementing the stream and transforming Jardim Ângela into one of the most densely populated districts of Sao Paulo.

All other members of IFDP were born in the periphery. Most of them are from the neighborhood where the ecovillage is located. Their life stories reflect the economic difficulties faced by their families (very often single mothers) in bringing up the children with low-paid jobs, and the close proximity to crime and violence. When they were growing up, there were different criminal gangs that fought against each other, and they grew up surrounded by violence and death.

In the 1990s, Rubens’ sons created a musical band together with some friends. They started using music to bring people from the neighborhood together, such as monthly parties to celebrate people's birthdays, music classes for the kids, and a recording studio. Because of their social work, in 2009, they were invited to visit Tamera, an ecovillage in Portugal. In Tamera, they were exposed to renewable energy, vegetarian cooking, and emotional sharing, and decided to bring all these technologies and practices to their slum. The members of the band decided to leave their jobs and dedicate themselves to creating an ecovillage. In 2010, they founded IFDP in Rubens and his family's house.

It was in this house that they carried out all of their activities. Over time, they created an off-grid solar energy system, an intelligent cistern that catches rainwater, a solar pump that takes the water to the toilet on the roof, and a biogas system that turns organic waste into gas for the stove of their community kitchen. Instituto Favela da Paz has recently managed to buy, through donations, the neighboring house and another plot of land where they will build their new headquarters. While ecovillage members raise funds for building the new headquarters, they were granted a 25-year lease on a big shed located near the plot. That is where many of their musical events now take place.

Instituto Favela da Paz today has 27 people living and working there, plus three people who work but do not live in the ecovillage. They also collaborate with a wide range of actors and networks. The only actors with whom they refuse to collaborate are political actors, of whom they express great distrust. This distrust comes from the fact that some local political actors have, in the past, tried to use the ecovillage to promote their own personal image toward their electorate (such as posting photos on social media), despite the fact that they had not supported the ecovillage in any way. Members of IFDP also feel that they are “filling in” for the absence of the government in their district. On several occasions, we heard people there saying that it was the government that was supposed to be doing the work they are doing. They also avoid discussing politics with participants and visitors.

During our research, we identified several ways through which IFDP promotes just energy transitions. Although some of them would not be considered energy-related by the dominant frameworks, the DEF helps us understand them in a different way. Many social and cultural activities of IFDP are energy-related because they reframe the narratives that keep in place the exploitation of cheap labor, especially from racialized groups. Instituto Favela da Paz's activities promote practices and narratives that challenge the “hold politics” by reclaiming a dignified existence and a share of the world for slum-dwellers through the reconstruction of cultural belongings and collective forms of living (*aquilombamento*).

Due to the limits of this article, we focus on four main ways through which IFDP promotes energy transitions: (1) the opening of the ecovillage as a “breathing space” in the slum, helping slum-dwellers to reframe their experiences and restore their agency; (2) their projects in the field of community-owned solar energy technologies, by which they disseminate and democratize decentralized energy technologies; (3) the reframing of meanings associated with time, human energy and money through their income-sharing practices, by which they question the role of peripheral actors as energy resources in the capitalist system; and (4) the reframing of meanings associated with Black identities, by which they question the historical role of Black actors as “body energy” resources.

### The Ecovela as a quilombo: “there is a place to breathe, to talk, to think, to learn”

Vini, one of the founders of IFDP, calls their project an “ecovela” (ecovillage + favela). The ecovillage develops different projects and events, such as women's groups, classes and workshops on vegetarian cooking, art, sustainable technologies, percussion, and audiovisual production, all free of charge. However, their most important goal is to provide spaces for participants to share and reframe their experiences of the world. They welcome people from all walks of life and provide a safe space for meaningful interactions, a “breathing space” as Vini puts it, where people can reflect on their own lives, their purpose, and their relationships. “*Because we have Favela da Paz there, we can breathe a little. […] There is another option for children, for young people, for older people. There is a place to breathe, to talk, to think, to learn”* (EI09-Vini, Pos. 2).

Alice, a resident of the ecovillage, gives an example of this. During one of her classes, a teenage boy arrived running, desperate, and very tense. He had been approached by the police who had threatened to kill him. When he told the group what had happened, there was a feeling of revolt and impotence. She then opened the space for people to share their feelings and even curse the police in a safe space. Then, they began to try to understand why the police officers acted the way they did and attempted to find a feeling of compassion:

So how do we give voice to these issues that are so strong, that are so heavy that sometimes it ends with a boy's destiny? Because it's a trauma like that which makes him rebel and say: “I wasn’t doing anything, now I’m going to do it. I’m going to get involved in crime and now I’m going to kill a police officer.” So one thing leads to another, and next thing you know, you stole his destiny, you stole his path. And when we have these spaces to decant, to look, to slow down… (FP03-Alice, Pos. 20)

According to all the interviewed members, the most important work behind their activities is to strengthen community ties, heal traumas of violence, and create a space of trust and mutual empowerment. Kelly, a community leader from a nearby slum, gives an example of this. She was invited to participate in a dance at IFDP for the campaign One Billion Rising, to bring visibility to violence against women. So Kelly invited a woman from her slum to come with her. After the dance, this woman told her that this event changed her life:

It transformed [her life] because she was beaten by her husband at home, her husband wouldn't let her have a cell phone. So she realized that she is a woman, that she does not deserve this type of treatment, this type of violence. […] And I think this is a very important thing, you know? Transform the mind […]. Now she doesn't accept her husband beating her, and before she accepted it. Would she have this image if one day she hadn’t gone to Favela da Paz and started thinking? (FP11-Kelly, Pos. 37-43)

Many of the interviewed external actors (neighbors and partners of IFDP) have reported that, at IFDP, they feel that they can be more honest, more open, and they can look for support in the people there. The doors of the ecovillage are always open. Those who want to participate in IFDP's activities are free to choose what projects they want to engage with, and therefore, have the space and time to discover the things they like to do. Instituto Favela da Paz also creates content for YouTube, such as their podcast *Mestres das Kebradas,* in which they interview “masters from the slum,” that is, musicians, teachers, and other local people who contribute to the community in some way.

And this shines in the eyes of the people around us. People feel valued, they feel: “well, you looked at me, you are here.” […] Because our work […] is to change people from the inside. […] I believe we've changed the lives of many people. And our lives too. […] There were many projects that emerged in the periphery where people have discovered themselves as a photographer, as a musician, as a journalist. (EI09-Vini, Pos. 4)

While members and participants of IFDP do not refer to the ecovillage as a *quilombo*, by analyzing their work through the DEF, we suggest that members of IFDP do promote dynamics of *aquilombamento*. They do so by creating a space for people who are marginalized by society to connect with one another and to restore collective bonds. They challenge the “hold politics” by reframing their experiences and helping to restore their agency for the creation of a World-Ship.

### Sustainable periphery: “but we see that discussions of environmental issues do not get to the periphery”

Sustainable periphery (in Portuguese *Periferia Sustentável*) is IFDP's project that focuses on low-cost sustainable technologies. One of the founders of the ecovillage, even without college education, has created a lab at the institute as well as a mobile lab, a solar-powered van which was refitted as a laboratory for workshops and other educational interventions. He uses this mobile lab to present sustainable technologies and to organize repair cafés for groups in different cities and different parts of Sao Paulo that would otherwise not have access to them.

Through the topic of sustainable periphery, IFDP started collaborating with a multinational engineering firm called Worley. Amanda is a trainee at Worley. She also lives in a peripheral district in the East Zone of the city. She had already noticed that discussions on environmental issues did not arrive in these peripheral regions. Inside the company, there is an Innovation Hub, where any employee can present ideas. So, in 2019, she decided to submit a proposal for her idea of installing solar panels in low-income communities.

After her proposal was approved, she found IFDP through a search on the internet. The first project they did together was the installation of Sao Paulo's first microgenerator of solar power, on the roof of the first house of the ecovillage. Worley financed the purchase of 22 solar panels of 410 W. Carrying out the project was not easy, as IFDP had a lot of late energy bills and no money to pay them all. In order to install the panels, all the bills had to be up to date. At the last minute, they received an anonymous donation, which allowed them to regularize their situation and carry out the project.

Worley also funded a course to train 22 solar panel installers (including six women), all from low-income backgrounds, who also received personal protection equipment. At least two of them have already succeeded in getting work in the field. There were many challenges, and it took months for the installation to be completed. Once completed, the project had a lot of coverage in mainstream media, which sparkled the interest of other community leaders from the periphery:

Well, we got the visibility we wanted. We got the population's interest in regard to solar panels, in regard to the course [of panel installers]. […]. And other [community] leaders come to me to find out more. We see that some projects are popping up in Sao Paulo […] so the objective has been achieved. People are interested, discussions are happening […] people want to take it to the places where they live. (FP08-Amanda, Pos. 59-63)

Now, IFDP and Worley are working on a follow-up project in collaboration with the Australian embassy in Brazil. The goal is to create a community energy project involving 30 families from Jardim Ângela. The Australian embassy donated 110 solar panels. However, they had some challenges related to the documentation of the shed where the panels were to be installed. The embassy requested formal documentation in order to carry out the project, including the receipts related to the payments of IPTU (the municipal tax on urban land use), which had not been paid for many years.

What happens is that most Brazilian [peripheral] communities were built like this, here where I live, most of the land is invaded, now that they're doing an urban readjustment […]. So for them [the embassy], this was a problem and ended up delaying things. (FP08-Amanda, Pos. 89)

The greatest challenge now is to unite the families in the legal form of an association. This will be the first grassroots community energy project in the city.

Through the sustainable periphery project, IFDP promotes just energy transitions by disseminating and democratizing renewable energy technologies. By doing so, they promote the insertion of racialized and marginalized people in environmental discussions, bridging the fracture that exists between environmental and antiracist movements (Ferdinand, 2022). While the creation of a community energy association is aligned with IFDP's commons-oriented practices, the role of a capitalist multinational company from the hydrocarbons sector may seem contradictory. We discuss this issue in discussion section.

### Income-sharing: “let's talk first about how we're going to donate ourselves”

The IFDP resident Lucas said to the first author one day: “*Sometimes I wonder if slavery is really over*” (Day20-Nov25, Pos. 19). He talked about why he thinks the capitalist system is crazy: people from the periphery wake up early, get onto a bus packed with people, go to a job they often don’t like, where 8 hours of their day is at the complete disposal of their bosses, get back onto another packed bus to a segregated part of the city, where there are few opportunities for leisure and cultural activities. They get home late and tired. The money they earn barely allows them to survive. The reflection he brought up is: is this very different from slavery?

Challenging the common practice of commuting to central regions for work, the ecovillage has developed an alternative model for dealing with work and money in which people's dreams and motivations are the primary driver. All residents work mainly (and many work only) at the ecovillage's projects and activities. All their courses and activities are offered free of charge. Their financial resources come from: (1) the concerts the band does, (2) donations, (3) artists that have the means to pay for the use of the studio and equipment, and (4) members who do external jobs and can donate part of their income to the communal pot. Sometimes they also submit project proposals to get public funding. However, this does not happen often because public grants demand a very complex project and financial accountability that is difficult for them to follow. All the money they receive goes to a common pot, and they decide together who or which project needs more money at that moment.

The most interesting aspect about this shared economy is that it is a system based on trust. There is no tracking of working hours. There is no comparison about who worked more or better than the other. Each one is engaged in one or more projects, and they receive according to their needs. Young people that arrive at the ecovillage and want to be a part of it are given time and space to participate in different activities until they figure out where they want to engage more, or even come up with new projects. Internal motivation is the main driver for their work, and this is a core value of the ecovillage.

Nevertheless, the growth of the institute in terms of the new headquarters and new people expected to join in the future brings many challenges regarding the maintenance of their shared economy. Bringing more people into the project creates difficulties because those that live in the ecovillage share everything. These new people, however, would probably need to be hired in a regular salary-based system. IFDP members are struggling with finding a solution to deal with this growth while maintaining their core values.

Because of this shared economy, ecovillage residents have the time to invest their human energy in their own projects. Instead of a conventional job, people at IFDP can work with art in a space free of pressure and oppression:

We created several incredible projects there, with music, with art, culture, with healthy food […], but it is only for us to be together, […] for us to keep simplicity, for us to keep the essence of what it is to be human. So that is why Favela da Paz is there. It can grow, but it will always maintain this idea. We want to know how the bacteria live inside the biogas system without receiving a salary. That's what we want to know, how can we do that. (EI09-Vini, Pos. 2)

The ecovillage, by doing things differently, stimulates reflection on the dominant ways of dealing with money. One of their neighbors, who is also a close partner of the ecovillage, talks about his learnings at the institute:

I think sharing was the best learning I could have here, to be more human out there, because here we have this all the time: “let's think about the other” […]. Money in here has no strength. What has strength is us being what we are, that's where the power lies. In other places the money comes first. We eliminate money here, completely. Everything we're going to talk about, let's talk first about how we're going to donate ourselves. (FP01-Jorge, Pos. 40, 46)

Through their income-sharing practices, IFDP is creating new narratives about time, energy, and work. They question the “hold politics” that confine Black bodies to the role of energy resources in the capitalist system. Instead of dedicating their time and energy to a low-paid job that produces benefits which will not be shared with them, they invest their time and energy in projects that are personally fulfilling and which generate shared benefits for the people around them. Through this commons-oriented practice, they become agents in the construction of a World-Ship.

### Reframing black identity: “my hair is my crown”

Art is one of the main foci of the ecovillage. They are also part of an artistic-cultural network that involves all of Sao Paulo, through which they collaborate with other cultural organizations from the peripheries. Instituto Favela da Paz and other actors from this network engage in bringing visibility to the Afro-Brazilian culture and movements of resistance, such as the samba, hip-hop, as well as musicians and poets from the periphery. At IFDP, they teach percussion, organize music events, record local artists, produce podcasts, and host a monthly open mic event (*sarau*). Members of IFDP open spaces for Black and peripheral people to see themselves as cultural producers. One of these spaces is the weekly rap battle, which attracts many young people from the district and from other peripheral areas. The ecovillage also gives local artists opportunities to record in their studio and to play at their concerts. At the shed, there are some big paintings on the walls from a local artist portraying female samba singers from the region. During events at the shed, the ecovillage offers spaces for locals to sell food and handicraft. We can say these cultural activities are commons-oriented because ecovillage members share their energy, their time, and their resources with the broader peripheral community where they live.

Not only music bridge the groups from this network. There are also common values and principles, such as the valorization of the Black body and identity; the honoring of the Black influence in Brazilian art, music, and dance; and the appreciation of the cultural resistance of the African diaspora in the Americas. Keto, from the collective Espírito de Zumbi, talks about his relationship with IFDP:

And then we started to realize that we had a lot in common. What we were doing, which is working for the community, which is making this transformation of the state of consciousness, many times of being just a kid from the periphery without much opportunity or projection, of wanting to be something, or do something, right? And we were making this opportunity happen. (FP09-Keto, Pos. 39)

The first author had the opportunity to participate in an event organized by IFDP called Festival Unikebradas, in which many of their collaboration partners participated. There were different round tables in which they discussed the richness of Black culture and ancestry. At one of these round tables, Keto said “*our ancestry is not in the slave quarters, it is in Africa and in the* quilombos.” One young local poet recited a poem that he wrote called *Self-esteem*. It talked about how long it took him to accept his own color, his own hair. Only at the age of 18 did he start accepting himself: “*My hair is my crown*” (Day28-Dec3, Pos. 40).

Through these activities, IFDP and other groups are rediscovering their bodies, their rhythms, and the myths about their own origins. As a result of their reframing efforts, Black bodies are seen as cultural creators, as artists, as belonging to a rich cultural tradition, and as empowered agents in a shared world.

## Discussion

Energy transitions take place against the backdrop of preexisting processes of uneven spatial development (Golubchikov and O'Sullivan, 2020; O'Sullivan et al., 2020). In the case of urban peripheries in Sao Paulo, this study has shown some of the difficulties of local populations in paying energy bills, their lack of money to invest in solar panels, and the often nonregularized situation of many of the houses. All of these factors prevent them from benefitting from decentralized renewable technologies. Peripheral urban areas also have less access to knowledge about energy transitions, showing how transitions may leave unquestioned historical processes of socioeconomic and political marginalization (Bringel and Fernandes, 2024).

Favela da Paz is a grassroots project created by slum-dwellers. Through the creation of networks and collaborations, IFDP is leading a transition initiative that focuses on energy generation *by and for the periphery.* This goes against the historical tendency of having peripheral places as sites of energy extraction in order to fuel the high-energy demands of the core, closely linking their project to the concept of energy justice. By including marginalized groups in environmental discussions, IFDP is promoting a “politics of encounter” in which those who had been relegated to the hold of the slave-ship can now participate in the building of a common World-Ship (Ferdinand, 2022).

When thinking of energy peripheries also as sites of exploitation of human body energy in the form of cheap labor, we can say that IFDP promotes energy transitions by stimulating the use of human energy *by and for the periphery.* Through their shared economy, IFDP supports a transition from an energy system based on extracting surplus energy from Black bodies to raise the standards of living of their bosses (Fiori, 2020), to a system in which human energy is invested in commons-oriented projects that benefit local communities and are based on convivial and emancipatory values.

By reframing meanings associated with work, time, and energy, IFDP's vision goes beyond labor-oriented approaches to just transitions based on demands for better wages, green jobs, and recognition in policies. Instituto Favela da Paz's members act from a position other than wage-workers, questioning the very necessity of exploitative relations. They converge, therefore, with the proposal of a just transition that can redesign the productive system according to workers’ autonomous needs, including giving them a chance to opt out of wage dependency (Velicu and Barca, 2020).

Similar to the community project studied by Lai (2023), members of IFDP are creating their own placed-based visions for sustainability. These visions go beyond renewable energy systems to also include the goal of restoring the agency and dignity of slum-dwellers, and their collective bonds. Instituto Favela da Paz and some of its partners can, therefore, be understood as *quilombos*, as defined by Nascimento (1980):

Quilombo […] means fraternal and free reunion, or encounter; solidarity, living together, existential communion […] complete fulfilment and realization of the creative capacities of the human being. All basic factors and elements of the economy are of collective ownership and use. Work is not defined as a form of punishment, oppression or exploitation; work is first a form of human liberation, which the citizen enjoys as a right and a social obligation (p. 161).

While ecovillage members do not use the concept of *quilombo*, their narratives, everyday practices, and philosophy do support this analytical categorization. Specifically, the following elements found at IFDP support this: (1) The engagement of marginalized and racialized groups in creating other ways of living together and relating to each other, by forming a space for mutual support; (2) Their efforts in maintaining and valuing Black culture and Black artists; (3) Their efforts to recreate collective forms of living, including collective ownership and shared economy. Through *aquilombamento*, IFDP and other similar organizations in the peripheries are creating spaces where people can question the colonial inhabiting, and where different ways of living and thinking can emerge.

However, as Ferdinand (2022) reminds us, the utopia of the *quilombo* could not take place completely outside the colonial world. Some of their material necessities were still dependent on the plantation. This dependence helps us understand the paradoxical collaboration between IFDP, whose practices are aligned with post-capitalist imaginaries (Nelson, 2024), and Worley, a capitalist multinational company. The collaboration with Worley is an indicator of the lack of public policies and trust in political actors that may lead grassroots projects to collaborate with the private sector. This collaboration is also an example of the importance of diversity in firms. This was a project born at the periphery of the company, as it was a trainee from the periphery who had the idea of taking solar energy projects to the slum.

Besides material necessities, a second limitation to *aquilombamento* is the fact that although these experiences do reveal paths of resistance, “the flight from the world does not change the world. […] Marronage did not bring about the end of slavery in the colonial world by itself or the overthrow of colonial inhabitation” (Ferdinand, 2022, pp. 157–158). Contemporary *quilombos* then face the same diffusion challenges of other grassroots innovations: “Paradoxically, a key benefit of grassroots innovations, namely, the ‘world within a world’, undermines diffusion. Whilst practices where “the rules are different” have certain strengths, those strengths become barriers when in concerted opposition to incumbent regimes” (Seyfang and Smith, 2007, p. 597).

In the specific case of ecovillages, the fact that most of them tend to be created in rural areas has been seen by some critics as isolating them from society and limiting their social impact (Dias et al., 2017). Tamera, the ecovillage in Portugal which inspired IFDP, for example, has created a “commons-based alternative political ecology” (Esteves, 2017, p. 989). However, this “commoning” process has also led to the creation of a “borderland”: a spatial, cultural, ecological, and socioeconomic estrangement from the local population (Esteves, 2017). In this sense, IFDP is different from other ecovillages. The fact that its founders were born in the slum, its opening to residents of the periphery with free cultural activities, as well as its networks with other peripheral groups of Sao Paulo, have greatly reduced the boundaries between ecovillage members and the surrounding population.

A recent study has shown that one way in which grassroots innovations such as ecovillages can influence broader society is through reframing, that is, embedding new frames and narratives for interpreting the world (Roysen et al., 2024). Similar to the dynamics of social movements, these new frames and narratives can, in turn, change the cultural landscape and the dominant narratives of a certain time (Jasper, 1997; Polletta, 1998; Strang and Soule, 1998; Whittier, 2007). In the case of IFDP, ecovillage members were not only creating new narratives within the ecovillage but also sharing them with neighbors and other groups from their territory. Their cultural work is not isolated but it is a node on a larger network of grassroots initiatives from the peripheries that are reframing meanings associated with the role and agency of peripheral actors. By presenting a “positive Black, periphery cultural identity,” this growing cultural movement from Sao Paulo's margins is “flipping” the racist and classist images usually associated with the periphery youth (Klein, 2019, p. 148).

Including these peripheral voices in discussions on just energy transitions is essential for building a World-Ship, and this is certainly a fruitful avenue for further research. Engaging with these prefigurative movements offers the possibility of repoliticizing energy research and creating transitions “not just to different energy systems but to different ways of living and relating” (Partridge, 2022, p. 81). Based on lived experiences of injustice and on place-based ethics, these situated knowledges also open up new understandings of justice from the ground up (Tornel, 2023).

## Conclusions

This article analyzed a grassroots initiative from the peripheries of the Global South through the lenses of the DEF. Instituto Favela da Paz is an urban ecovillage (an “ecovela”) created by people that live in a situation of socioeconomic and civic vulnerability but who were able to reframe their experiences and develop different innovation projects.

This study contributes to critical scholarship on just energy transitions in two ways: Firstly, by documenting a case study of a decolonial and commons-oriented project that is shaping energy transitions from below. Secondly, by bringing to the forefront the exploitation of human body energy—be it in the form of slavery, cheap labor, gender inequality, or others—that maintains dominant and unjust patterns of energy production, distribution, and consumption. If current carbon pollution is mainly caused by social inequalities, a just energy transition requires a transformation of the acute social and economic pyramids. This would, necessarily, entail a transition toward a low-energy-consumption standard of living for all humans and a consequent reduction in the wealth of the rich. What is at stake is not whether the renewable energy sector creates job opportunities for marginalized communities, but whether marginalized communities have the agency to create their own visions of sustainability and to live meaningful and fulfilling lives.

The DEF invites us to reimagine the ways in which we can create a sustainable future by suggesting the image of a World-Ship in which there is no “hold”: a way of inhabiting the Earth in which no human group should be reduced to the role of human energy production for the benefit of a few. Future studies should, therefore, consider the transformation of current modes of exploitation of human energy as part of a just energy transition. This could comprise, for example, the creation of commons-oriented and cooperative forms of enterprise in all sectors of society. More attention should be paid to the narratives and place-based visions for sustainability that are emerging in the peripheries, as they may offer transition scholars and practitioners alternative ways to conceive socially just pathways toward energy transitions. Discussions on energy transitions cannot take place exclusively among those who benefit the most from cheap externalized labor and cheap fossil fuels. The profound transformations needed for a just energy transition require the inclusion of the voices and visions for the future of those who have been silenced until now.

## Notes

1. In Brazil, the concept of ecovillages has been linked to intentional communities of neo-rurals: people from upper-middle classes who leave the cities and go to rural areas to develop alternative and more sustainable lifestyles. In this sense, Instituto Favela da Paz differs from more common formats of ecovillages in the country due to the socioeconomic background of its members.

2. In the decolonial ecology framework, “world” refers to the multiple social and political relationships among the plurality of humans and non-humans, the political (and embodied) action of living together.

3. The focus group for the discussion of results was carried out online, and only two people managed to participate: Lucas (resident) and Kelly (external partner). We also shared a translated version of the manuscript with the ecovillage and interviewed partners for feedback before submitting it.
